# Structure–activity relationship investigation of benzamide and picolinamide derivatives containing dimethylamine side chain as acetylcholinesterase inhibitors

**DOI:** 10.1080/14756366.2017.1399885

**Published:** 2017-11-22

**Authors:** Xiao-hui Gao, Lin-bo Liu, Hao-ran Liu, Jing-jing Tang, Lu Kang, Hongnian Wu, Peiwu Cui, Jianye Yan

**Affiliations:** a Key Laboratory Breeding Base of Hu’nan Oriented Fundamental and Applied Research of Innovative Pharmaceutics, College of Pharmacy, Changsha Medical University, Changsha, China;; b College of Chemistry and Chemical Engineering, Hu’nan University, Changsha, China;; c College of Pharmacy, Hu’nan University of Chinese Medicine, Changsha, China

**Keywords:** Cholinesterase inhibitors, benzamide, picolinamide, structure–activity relationship, Alzheimer's disease

## Abstract

A series of benzamide and picolinamide derivatives containing dimethylamine side chain (**4a**–**4c** and **7a**–**7i**) were synthesised and evaluated for acetylcholinesterase (AChE) and butyrylcholinesterase (BChE) inhibitory activity *in vitro*. Structure–activity relationship investigation revealed that the substituted position of dimethylamine side chain markedly influenced the inhibitory activity and selectivity against AChE and BChE. In addition, it seemed that the bioactivity of picolinamide amide derivatives was stronger than that of benzamide derivatives. Among them, compound **7a** revealed the most potent AChE inhibitory activity (IC_50_: 2.49 ± 0.19 μM) and the highest selectivity against AChE over BChE (Ratio: 99.40). Enzyme kinetic study indicated that compound **7a** show a mixed-type inhibition against AChE. The molecular docking study revealed that this compound can bind with both the catalytic site and the peripheral site of AChE.

## Introduction

Alzheimer’s disease (AD), as a chronic and progressive neurodegenerative disorder characterised by memory loss, language impairment and intellectual ability degression, is one of most common diseases in the elderly population^1–3^. Although the precise aetiology of AD is not elucidated enough, AChE inhibitors remain the primary drugs for the therapy to increase acetylcholine level in brain to meliorate the symptom^4^^,^^5^.

In recent decades, many natural products or their derivatives were discovered as new potential remedy for the treatment of AD^6–9^. Followed by these investigations, a series of natural products derivatives were synthesised and evaluated for AChE inhibitory activity in our laboratory. Among them, Mannich base derivatives of Flavokawain B with chalcone structure were found better AChE inhibitory activity than other compounds^10^. Afterwards, a series of chalcone nitrogen-containing derivatives were synthesised and revealed potent AChE inhibitory activity^11–13^. All results suggested that two aromatic rings with a spacer were possible privilege structures for the design of AChE inhibitors.

**Figure 1.**
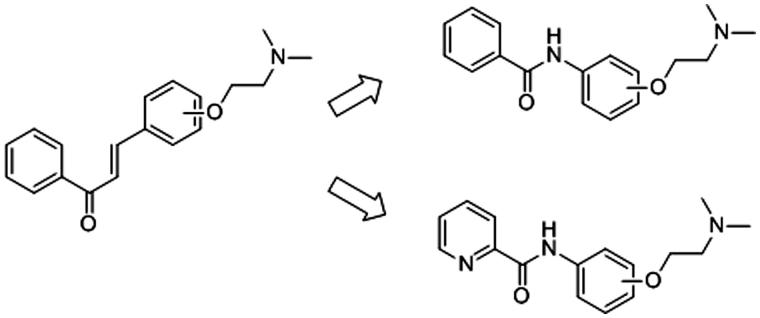
Comparison of chalcone derivatives and amide derivatives with diethanolamine side chain.

Here, we intent to explore whether the α,β-unsaturated carbonyl group linked two benzene rings in chalcone structure can be replaced by other structural units? And the substituted position of nitrogen-containing side chain can influence the inhibition activity against AChE? Thus, a series of benzamide and picolinamide derivatives were designed, synthesised and evaluated the biological activity of inhibiting AChE (Figure 1). These derivatives are similar to chalcone derivatives in our previous investigations. In fact, recently, a lot of benzamide and picolinamide derivatives were investigated as anticancer agents^14^, DNA minor groove binders^15^, 11 b-hydroxysteroid dehydrogenase inhibitos^16^ or metabotropic glutamate receptor 5 antagonists^17^, but few investigations on the bioactivity of them in inhibiting AChE or BChE were reported^18^. In order to study the possible inhibition profile and mechanism of new synthesised compounds, enzyme kinetic experiments and molecular docking studies were performed.

## Materials and methods

### Chemistry

All chemicals or reagents were of analytical reagent grade without further purification. The purity of compounds was checked by Shimadzu LC-20 A high-performance liquid chromatography. The melting points were measured on a WRS-lA melting point detector. Infrared spectra were obtained from Shimadzu Infinity-1 infrared spectrometer. ^1^H NMR spectra were obtained from a Bruker 400 MHz NMR spectra instrument in CDCl_3_ with TMS as the internal reference. Mass spectra were obtained from Finnigan LCQ Advantage MAX by electrospray ionisation (ESI-MS).

### General procedure for the synthesis of 3a–3c

Benzoyl chloride (compound **2**) was synthesised from benzamide (compound **1**, 5 mmol) with excess oxalyl chloride (2.55 ml, 30 mmol) in CH_2_Cl_2_ (40 ml) containing N,N-dimethylformamide (DMF) as a catalytist. The mixture was refluxed for about 2 h until the disappearance of the benzoyl acid monitoring by TLC, and then, the mixture was cooled to room temperature. The redundant oxalyl chloride was evaporated under vacuum. The crude benzoyl chloride was used in the following reaction without further purification.

The benzoyl chloride was dissolved in acetonitrile (20 ml) and then para-aminophenol, meta-aminophenol or ortho-aminophenol dissolving in toluene (1 ml, 11 mmol) was drop-wise added to the acetonitrile solution in an ice bath, respectively. The mixture was refluxed for 6–10 h until benzoyl chloride disappeared. After the solvent was removed under reduced pressure, the crude product was added into 30% sodium hydroxide solution and filtered, 10% HCl was applied to adjust pH of the solution to 3–4, followed by the production of the precipitation. Then light grey solid compound **3a**, **3b** and **3c** were gained with yield of 80–90%.

### General method for synthesis of 4a–4c

Compound **4a**–**4c** was synthesised from compound **3a–3c** (0.3208 g, 1.5 mmol) and 2-dimethylaminoethyl chloride hydrochloride (0.6729 g, 4.5 mmol) and purified using silica-gel column chromatography with methanol/dichloromethane (1:5 5 ∼ 70, v/v) as elution.

Spectrum data of compounds **4a**–**4c** for the characterisation are outlined in Supplement data.

### General procedure for the synthesis of 6a–6i

Hydroxybenzotriazole (HOBt) and dicyclohexylcarbodiimide (DCC) was added into an anhydrous toluene solution (15 ml) containing compound **5a**, **5b** or **5c** (5 mmol, 1.0 equiv) at 0 °C, and the mixture was stirred for 45 min. Then, a series of aminophenol (2-aminophenol, 3- aminophenol and 4-aminophenol) (5.5 mmol, 1.1 equiv) were added to the reaction solution, respectively. The reaction mixture was stirred at room temperature for 10–12 h until compound **5a**, **5b** or **5c** disappeared. Then, the reaction mixture was filtered and the toluene was evaporated under reduced pressure. The residue was dissolved in 10% NaOH and filtered, and then, 15% HCl was added to adjust pH of the solution to 3–4, followed by the production of precipitation. The desired products phenylcinnamide (compounds **6a**–**6i**) were gained from the solution by filtration with a good yield of 70 ∼ 75%.

### General procedure for the synthesis of 7a–7i

Compounds **7a**–**7i** were synthesised from compound **6a**–**6i** (1.5 mmol) and 2-dimethylaminoethyl chloride hydrochloride (4.5 mmol) with the general procedure and purified using silica-gel column chromatography with methanol/dichloromethane as elution to gain the desired products.

Spectrum data of compounds **7a**–**7i** for the characterisation are outlined in Supplement data.

### AChE and BChE inhibition assay

AChE/BChE activity assays were carried out by *Ellman* method with slight modification^19^. Each compound was dissolved in Tween 80 and diluted with water to various concentrations immediately before use. The assay solution which contained 40 μL AChE/BChE, 100 μL acetylthiocholine iodide/S-butyrylthiocholine iodide, 2.76 ml Na_2_HPO_4_/NaH_2_PO_4_ buffer (pH 8.0, 0.1 M), and 100 μL tested compound solution with different concentrations were incubated at 30 °C for 25 min. Then, the reaction was terminated with 100 μL 20% sodium dodecylsulphate (SDS), then 100 μL 10 mM 5, 5′-Dithiobis-(2-nitrobenzamide) (DTNB) as chromogenic agent was added into the mixture. The absorbance of each assay mixture was measured at 412 nm by UV spectroscopy, which showed a linear relationship with the activity of AChE/BChE. The IC_50_ values were calculated by Bliss method and expressed as Mean ± SD of the replicates.

### Kinetic studies

Kinetic characterisation of AChE/BChE was performed by a modified method previous reported^20^. Compound **7a** was pre-incubated with the enzyme at 30 °C, then 100 µL acetylthiocholine iodide including five concentrations was added into the assay mixture and kinetic characterisation was conducted spectrometrically at 412 nm. Additionally, the parallel control experiment was made without compound **7a** in the mixture.

### Molecular docking

Molecular modelling was performed by Molecular Operating Environment (MOE) software package. The X-ray crystallographic structures of AChE (PDB code: 1EVE)^21^ and BChE (PDB code: 1P0I)^22^ were gained from protein data bank. 3 D structure of compound **7a** was established, and docked into the active site of the protein after energy being minimised. The dock scoring in MOE software was done by ASE scoring function.

### Log *p* measurement

Octanol–water partition coefficients of compounds **4a**–**4c**, **7a∼7i** were measured by the shake flask method described previously^23^. The aqueous phase was replaced by phosphate buffer solution (PBS, pH = 7.4). The assay mixture containing tested compounds was shaken at 37 °C over night and then centrifuged at 2000 rpm for 20 min, followed by the analysis with HPLC. A C_18_ column (150 nm × 4.6 mm, 5 μm) was used with the mobile phase of methanol-0.1% triethylamine (TEA) (85:15, v/v) at a flow rate of 1.0 ml.min^−1^ and the detection wavelength of 283 nm at 32 °C. Experiments were conducted in triplicate and log *p* values were calculated.

## Results and discussion

### Chemistry

**Scheme 1.**
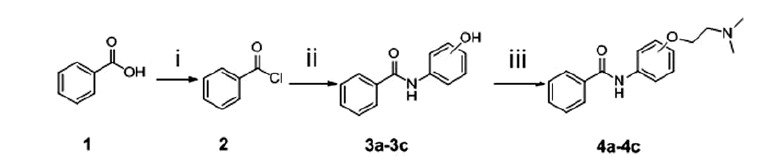
Synthesis of benzamide derivatives. Reagents and conditions: (i) (COCl)_2_, DCM, reflux;(ii) aminophenol, TEA, acetonitrile, 60 °C; (iii) (CH_3_)_2 _N(CH_2_)_2_Cl_2_, K_2_CO_3_, NaI, acetone, reflux.

**Scheme 2.**
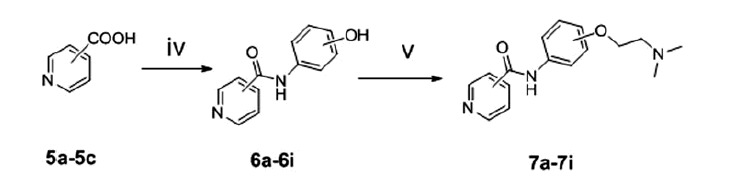
Synthesis of picolinamide amide derivatives. Reagents and conditions: (iv) aminophenol, DCC, HOBT, toluene, RT; (v) (CH_3_)_2 _N(CH_2_)_2_Cl_2_, K_2_CO_3_, NaI, acetone, reflux.

The synthetic routes of benzamide derivatives (compound **4a**–**4c**) and picolinamide derivatives (compound **7a**–**7i**) are outlined in Schemes 1 and 2. First, benzamide or picolinamide reacted with a series of amines (*p*-aminophenol, *m*-aminophenol, *o*-aminophenol) in the presence of oxalyl chloride and TEA in dichloromethane to gain compounds **3a**–**3c**, respectively. Then chloroethyldimethylamine in acetone was applied to generate compounds **4a**–**4c** in the presence of K_2_CO_3_ and NaI. The final products were purified by silica gel chromatography and characterised by ^1^H NMR, IR and MS. The synthesis pathway of these two series compounds had a little difference. Benzamide can react easily with oxalyl chloride and gain benzoyl chloride with an excellent yield, but no perfect products gained to synthesise picolinamide amides from picolinamide using this method, so another pathway was used to generate picolinamide amides with DCC, HOBT as catalysts.

### Structure–effect relationship of new synthesised compounds on the inhibition against AChE and BChE

The half maximal inhibitory concentration (IC_50_ values) of new synthesised compounds for AChE and BChE as well as the selectivity for AChE were summarised in Table 1. It indicated that the alteration of substituted position of dimethylamine significantly influenced the activity and the selectivity of compounds. Among them, the inhibitory potency and selectivity of benzamide and picolinamide amide derivatives against AChE was ranked by the following order: Para-substituted dimethylamine > meta-substituted dimethylamine > ortho-substituted dimethylamine. However, interestingly, the inhibitory potency of almost compounds against BChE was ranked by the order: ortho-substituted dimethylamine > meta-substituted dimethylamine > para-substituted dimethylamine. Among them, compound **7a** with IC_50_ values of 2.49 ± 0.19 µM, showed more potent than Rivastigmine (IC_50_ = 10.54 ± **0.26 **µM) In addition compound **7a** revealed the highest selectivity for AChE (Ratio: 99.40).

**Table 1.** Cholinesterase inhibitory activity and log *p* values of benzamide and picolinamide derivatives.

		IC_50_^a^ (μM) ± SD			
Compound	Structure	AChE	BChE	Selectivity^b^	Log *p*^c^
**4a**		18.22 ± 1.16	142.94 ± 5.56	7.84	1.12
**4b**		75.79 ± 4.13	20.91 ± 1.61	0.276	1.18
**4c**		>500	17.96 ± 1.00	<0.0359	1.23
**7a**		2.49 ± 0.19	247.50 ± 7.54	99.4	1.26
**7b**		38.45 ± 2.19	48.55 ± 2.23	1.26	1.31
**7c**		>500	44.58 ± 1.16	<0.0892	1.37
**7d**		5.76 ± 0.41	246.51 ± 1.85	42.80	1.33
**7e**		13.25 ± 1.73	22.15 ± 1.01	1.67	1.37
**7f**		75.79 ± 0.84	33.41 ± 1.37	0.441	1.42
**7g**		4.68 ± 0.30	114.36 ± 7.73	24.4	1.14
**7h**		72.98 ± 3.03	35.91 ± 0.94	0.492	1.37
**7i**		>500	27.43 ± 2.31	<0.0549	1.18
Rivastigmine		10.5 ± 0.86	0.26 ± 0.08	0.0247	–

a IC_50_ values of compounds represent the concentration that caused 50% enzyme activity loss.

b Selectivity for AChE is defined as IC_50_ (BChE)/IC_50_ (AChE).

c The partition coefficients of in the octanol/buffer solution at pH 7.4 were determined by the classical shake-flask method.

Log *p* values of new synthesised compounds were ranged from 1.12 to 1.42, which indicated that all the compounds are possible sufficiently lipophilic to pass the blood brain barrier (BBB) *in vivo*^24^.

Compound **7a** was selected for kinetic studies. The linear Lineweaver–Burk equation of the Michaelis–Menten was applied to evaluate the inhibition profile. The analysis of the steady-state inhibition data of compound **7a** was shown in Supplement data: Table 2. According to the analysis, *K*_m_ but not *V*_max_ increased with the increasing concentration of compound **7a**, which presented a mixed-type inhibition. The competitive inhibition constant (*K*_i_) and the non-competitive constant (*K*_i’_) are 4.69 μM and 3.28 μM, respectively.

Molecular docking was conducted for compound to study the possible inhibition mode with AChE or BChE. 3 D structure of compound **7a** was established by virtue of the builder interface of MOE program and docked into the active site of the protein after energy being minimised (For AChE, −22.7507 kcal.mol^−1^; For BChE, −16.6987 kcal.mol^−1^). As shown in Supplement data, compound **7a** exhibited multiple points binding modes with AChE (Supplement data: Figure 2 (A,B)). The binding points of compound **7a** with AChE were Trp84(3.92 Å), Trp279(4.12 Å) and Tyr334(3.89 Å) (Supplement data: Table 3), while the binding points with BChE was Tyr252(4.43 Å) (Supplement data: Figure 2(A,B); Table 3). For both of them, the conformation of the side chain conformed to the shape of the mid-gorge, but compound **7a** could not combine with Trp82 in BChE, which was an important amino acid in catalysis domain. The results above were possible to partial explain its potent and selective inhibition for AChE.

## Conclusions

In summary, a new series of benzamide and policnamide derivatives were designed, synthesised and evaluated for their effects on AChE and BChE. SAR investigation showed that all compounds with *Para*-substituted dimethylamine side chain had more potent inhibition activity and selectivity against AChE compared with *Meta*- or *Ortho*-substituted ones. In addition, the inhibition potency against AChE of picolinamide derivatives was more potent than that of benzamide derivatives. Based on these results earlier, it seems that an important rule is discovered and possibly applied in the future: we can alter the substituted position of nitrogen-containing side chain to gain selective and/or potent AChE inhibitors conveniently, which can be helpful for the development of potential AChE inhibitors for the treatment of AD.

## Supplementary Material

IENZ_1399885_Supplementary_Material.pdf
