# Supplementary material for: Structure–activity relationship investigation of benzamide and picolinamide derivatives containing dimethylamine side chain as acetylcholinesterase inhibitors
Source: J Enzyme Inhib Med Chem. 2017 Nov 22;33(1):110–4. doi: 10.1080/14756366.2017.1399885 (PMC6009985; doi:10.1080/14756366.2017.1399885)
Supplement: IENZ_1399885_Supplementary_Material.pdf [file IENZ_A_1399885_SM8567.pdf]

Supplemental data

1 **Spectrum data of compounds 4a-4c and 7a-7i**

1.1 N-(4-(2-(dimethylamino)ethoxy)phenyl)benzamide (4a)

A yellow solid product, yield 78%, mp: 148.3-149.2 ℃. ^{1}H NMR (400 MHz, DMSO-*d*$_6$) δ (ppm): 2.22 (6H, s, 2× NC$\underline{H}_3$), 2.60-2.63 (2H, t, *J* =12.0 Hz, OCH$_2$C$\underline{H}_2$), 4.01-4.04 (2H, t, *J* =12.0 Hz, OC$\underline{H}_2$CH$_2$), 6.92-6.94 (2H, d, *J* =8.0 Hz, Ar-H), 7.56-7.60 (2H, m, Ar-H), 7.56-7.60 (1H, m, Ar-H), 7.65-7.67 (2H, d, *J* =8.0 Hz, Ar-H), 7.93-7.95 (2H, d, *J* =8.0 Hz, Ar-H), 10.12 (1H, s, N$\underline{H}$). IR (KBr) m/cm^{-1}: 3315, 3034, 1661, 1549, 1516, 1261, 1184. MS m/z (ESI): 285 [M+H]+. Purity: 99.8% by HPLC (MeOH/0.1% TEA 85:15 (v/v); t$_R$(min):2.15.

1.2 N-(3-(2-(dimethylamino)ethoxy)phenyl)benzamide (4b)

A white solid product, yield 74%, mp: 72.3-73.5 ℃. ^{1}H NMR (400 MHz, DMSO-*d*$_6$) δ (ppm): 2.23 (6H, s, 2× NC$\underline{H}_3$), 2.62-2.65 (2H, t, *J* =12.0 Hz, OCH$_2$C$\underline{H}_2$), 4.02-4.05 (2H, t, *J* =12.0 Hz, OC$\underline{H}_2$CH$_2$), 6.67-6.70 (1H, m, Ar-H), 7.22-7.26 (1H, m, Ar-H), 7.35-7.37 (1H, t, *J* =8.0 Hz, Ar-H), 7.48 (1H, s, Ar-H), 7.51-7.55 (2H, m, Ar-H), 7.58-7.61 (1H, t, *J* =12.0 Hz, Ar-H), 7.93-7.95 (2H, d, *J* =8.0 Hz, Ar-H), 10.19 (1H, s, N$\underline{H}$).

IR (KBr) m/cm^{-1}: 3321, 3016, 1647, 1598, 1541, 1259, 1182. MS m/z (ESI): 285 [M+H]+. Purity: 99.6% by HPLC (MeOH/0.1% TEA 85:15 (v/v); t$_R$(min):2.30

1.3 N-(2-(2-(dimethylamino)ethoxy)phenyl)benzamide (4c)

A yellow oil product, yield 72%, ^{1}H NMR (400 MHz, DMSO-*d*$_6$) δ (ppm): 2.20 (6H, s, 2× NC$\underline{H}_3$), 2.59-2.62 (2H, t, *J* =12.0 Hz, OCH$_2$C$\underline{H}_2$), 4.24-4.27 (2H, t, *J* =12.0 Hz, OC$\underline{H}_2$CH$_2$), 7.10-7.14 (1H, m, Ar-H), 7.19-7.23 (1H, m, Ar-H), 7.26-7.28 (1H, d, *J* =8.0 Hz, Ar-H), 7.62-7.65 (2H, t, *J* =12.0 Hz, Ar-H), 6.67-7.71 (1H, m, Ar-H), 7.98-8.00 (2H, d, *J* =8.0 Hz, Ar-H), 8.18-8.20 (1H, d, *J* =8.0 Hz, Ar-H), 9.99 (1H, s, N$\underline{H}$). IR (KBr) m/cm^{-1}: 3350, 3026, 1585, 1539, 1498, 1195, 1195.MS m/z (ESI): 285 [M+H]+. Purity: 98.9% by HPLC (MeOH/0.1% TEA 85:15 (v/v); t$_R$(min):2.52

1.4 N-(4-(2-(dimethylamino)ethoxy)phenyl)isonicotinamide (7a)

A yellow solid product, yield 62%, mp: 103.5-105.1. ^{1}H NMR (400 MHz, DMSO-d_6) δ (ppm): 2.26 (6H, s, 2× NCH_3), 2.66-2.69 (2H, t, J =12.0 Hz, OCH_2CH_2), 4.04-4.07 (2H, t, J =12.0 Hz, OCH_2CH_2), 6.95-6.97 (2H, d, J =8.0 Hz, Ar-H), 7.66-7.68 (2H, d, J =8.0 Hz, Ar-H), 7.84-7.86 (2H, d, J =8.0 Hz, pyridine-H), 8.77-8.79 (2H, d, J =8.0 Hz, pyridine-H), 10.40 (1H, s, NH). IR (KBr) m/cm^{-1}: 3325, 3031, 1500, 1544, 1465, 1178, 1165. MS m/z (ESI): 286 [M+H]+. Purity: 98.9% by HPLC (MeOH/0.1% TEA 85:15 (v/v); t_R(min). 2.33

1.5 N-(3-(2-(dimethylamino)ethoxy)phenyl)isonicotinamide (7b)

A yellow oil product, yield 75%, ^{1}H NMR (400 MHz, DMSO-d_6) δ (ppm): 2.33 (6H, s, 2× NCH_3), 2.78-2.81 (2H, t, J =12.0 Hz, OCH_2CH_2), 4.08-4.11 (2H, t, J =12.0 Hz, OCH_2CH_2), 6.72-6.74 (1H, m, Ar-H), 7.25-7.29 (1H, t, J =16.0 Hz, Ar-H), 7.34-7.36 (1H, d, J =8.0 Hz, Ar-H), 7.57 (1H, s, Ar-H), 7.84-7.86 (2H, d, J =8.0 Hz, pyridine-H), 8.77-8.79 (2H, d, J =8.0 Hz, pyridine-H), 10.40 (1H, s, NH). IR (KBr) m/cm^{-1}: 3331, 3018, 1600, 1595, 1544, 1184, 1165. MS m/z (ESI): 286 [M+H]+. Purity: 98.4% by HPLC (MeOH/0.1% TEA 85:15 (v/v); t_R(min). 2.517

1.6 N-(2-(2-(dimethylamino)ethoxy)phenyl)isonicotinamide (7c)

A yellow solid product, yield 72%, mp: 108.3-112.0.^{1}H NMR (400 MHz, DMSO-d_6) δ (ppm): 2.13 (6H, s, 2× NCH_3), 2.53-2.56 (2H, t, J =12.0 Hz, OCH_2CH_2), 4.16-4.18 (2H, t, J =12.0 Hz, OCH_2CH_2), 7.04-7.06 (1H, m, Ar-H), 7.19-.20 (2H, m, Ar-H), 7.80-7.82 (2H, d, J =8.0 Hz, pyridine-H), 7.97-7.99 (1H, d, J =8.0 Hz, Ar-H), 8.80-8.82 (2H, d, J =6.0 Hz, pyridine-H), 10.18 (1H, s, NH). IR (KBr) m/cm^{-1}: 3327, 3028, 1647, 1597, 1541, 1182, 1078. MS m/z (ESI): 286 [M+H]+. Purity: 99.4% by HPLC (MeOH/0.1% TEA 85:15 (v/v); t_R(min). 2.75

1.7 N-(4-(2-(dimethylamino)ethoxy)phenyl)nicotinamide(7d)

A yellow solid product, yield 78%, mp: 105.5-105.8. ^{1}H NMR (400 MHz, DMSO-d_6) δ (ppm): 2.22 (6H, s, 2× NCH_3), 2.60-2.63 (2H, t, J =12.0 Hz, OCH_2CH_2), 4.02-4.05 (2H, t, J =12.0 Hz, OCH_2CH_2), 6.91-6.96 (2H, d, J =20.0 Hz, Ar-H), 7.55-7.57 (1H, m, pyridine-H), 7.65-7.67 (2H, d, J =8.0 Hz, Ar-H), 8.27-8.29 (1H, d, J =8.0 Hz, pyridine-H), 8.75-8.76 (1H, d, J =4.0 Hz, pyridine-H), 9.09-9.10 (1H, d, J =4.0 Hz, pyridine-H), 10.33 (1H, s, NH). IR (KBr) m/cm^{-1}: 3351, 3039, 1670, 1581,

1558, 1188, 1091. MS m/z (ESI): 286 [M+H]+. Purity: 98.3% by HPLC (MeOH/0.1% TEA 85:15 (v/v); t_R(min). 2.517

1.8 N-(3-(2-(dimethylamino)ethoxy)phenyl)nicotinamide (7e)

A yellow oil product, yield 71%, ^{1}H NMR (400 MHz, DMSO-d_6) δ (ppm): 2.33 (6H, s, 2× NC$\underline{H}_3$), 2.78-2.81 (2H, t, J =12.0 Hz, OCH$_2$C$\underline{H}_2$), 4.08-4.11 (2H, t, J =12.0 Hz, OC$\underline{H}_2$CH$_2$), 6.72-6.74 (1H, m, Ar-$\underline{H}$), 7.25-7.29 (1H, t, J =16.0 Hz, Ar-$\underline{H}$), 7.34-7.36 (1H, d, J =8.0 Hz, Ar-$\underline{H}$), 7.57 (1H, s, Ar-$\underline{H}$), 7.56-7.59 (1H, m, pyridine-$\underline{H}$), 8.29-8.31 (1H, d, J =12.0 Hz, pyridine-$\underline{H}$), 8.76-8.77 (1H, d, J =4.0 Hz, pyridine-$\underline{H}$), 9.10 (1H, d, J =8.0 Hz, pyridine-$\underline{H}$), 10.46 (1H, s, N$\underline{H}$). IR (KBr) m/cm^{-1}: 3316, 3031, 1668, 1627, 1600, 1188, 1097. MS m/z (ESI): 286 [M+H]+. Purity: 98.2% by HPLC (MeOH/0.1% TEA 85:15 (v/v); t_R(min).2.517

1.9 N-(2-(2-(dimethylamino)ethoxy)phenyl)nicotinamide (7f)

A yellow oil product, yield 76%, ^{1}H NMR (400 MHz, DMSO-d_6) δ (ppm): 2.13 (6H, s, 2× NC$\underline{H}_3$), 2.53-2.56 (2H, t, J =12.0 Hz, OCH$_2$C$\underline{H}_2$), 4.16-4.18 (2H, t, J =12.0 Hz, OC$\underline{H}_2$CH$_2$), 7.04-7.06 (1H, m, Ar-$\underline{H}$), 7.19-.20 (2H, m, Ar-$\underline{H}$), 7.56-7.59 (1H, m, pyridine-$\underline{H}$), 8.29-8.31 (1H, d, J =12.0 Hz, pyridine-$\underline{H}$), 8.76-8.77 (1H, d, J =4.0 Hz, pyridine-$\underline{H}$), 9.10 (1H, d, J =8.0 Hz, pyridine-$\underline{H}$), 10.46 (1H, s, N$\underline{H}$).IR (KBr) m/cm^{-1}: 3288, 3015, 1674, 1598, 1560, 1166, 1095. MS m/z (ESI): 286 [M+H]+. Purity: 98.5% by HPLC (MeOH/0.1% TEA 85:15 (v/v); t_R(min).2.00

1.10 N-(4-(2-(dimethylamino)ethoxy)phenyl)picolinamide (7g)

A yellow solid product, yield 65%, mp: 111.8-113.5.^{1}H NMR (400 MHz, DMSO-d_6) δ (ppm): 2.22 (6H, s, 2× NC$\underline{H}_3$), 2.61-2.64 (2H, t, J =12.0 Hz, OCH$_2$C$\underline{H}_2$), 4.02-4.05 (2H, t, J =12.0 Hz, OC$\underline{H}_2$CH$_2$), 6.93-6.96 (2H, m, Ar-$\underline{H}$), 7.65-7.69 (1H, m, pyridine-$\underline{H}$), 7.79-7.83 (2H, m, Ar-$\underline{H}$), 8.04-8.06 (1H, m, pyridine-$\underline{H}$), 8.14-8.16 (1H, d, J =8.0 Hz, pyridine-$\underline{H}$), 8.73-8.74 (1H, d, J =4.0 Hz, pyridine-$\underline{H}$), 10.56 (1H, s, N$\underline{H}$). IR (KBr) m/cm^{-1}: 3320, 3036, 1672, 1602, 1558, 1174, 1095. MS m/z (ESI): 286 [M+H]+. Purity: 98.5% by HPLC (MeOH/0.1% TEA 85:15 (v/v); t_R(min).1.92

1.11 N-(3-(2-(dimethylamino)ethoxy)phenyl)picolinamide (7h)

A yellow oil product, yield 68%, ^{1}H NMR (400 MHz, DMSO-d_6) δ (ppm): 2.23 (6H, s, 2× NC$\underline{H}_3$), 2.63-2.66 (2H, t, J =12.0 Hz, OCH$_2$C$\underline{H}_2$), 4.04-4,07 (2H, t, J =12.0

Hz, OCH_2CH_2), 6.69-6.72 (1H, m, Ar-H), 7.23-7.27 (1H, t, *J* =16.0 Hz, Ar-H), 7.50-7.52 (1H, d, *J* =8.0 Hz, Ar-H), 7.61-7.62 (1H, t, *J* =4.0 Hz, Ar-H), 7.67-7.70 (1H, m, pyridine-H), 8.06-8.10 (1H, m, pyridine-H), 8.16-8.18 (1H, d, *J* =16.0 Hz, pyridine-H), 8.74-8.75 (1H, m, pyridine-H), 10.60 (1H, s, NH). IR (KBr) m/cm^{-1}: 3339, 3105, 1664, 1600, 1583, 1174, 1078. MS m/z (ESI): 286 [M+H]+. Purity: 99.3% by HPLC (MeOH/0.1% TEA 85:15 (v/v); t_R(min).2.00

1.12 N-(3-(2-(dimethylamino)ethoxy)phenyl)picolinamide (7i)

A yellow oil product, yield 63%, ^{1}H NMR (400 MHz, DMSO-d_6) δ (ppm): 2.46 (6H, s, 2× NCH_3), 2.93-2.95 (2H, t, *J* =12.0 Hz, OCH_2CH_2), 4.27-4,30 (2H, t, *J* =12.0 Hz, OCH_2CH_2), 7.01-7.05 (1H, m, Ar-H), 7.10-7.18 (2H, m, Ar-H), 7.68-7.81 (1H, m, pyridine-H), 8.08-8.12 (1H, m, Ar-H), 8.18-8.20 (1H, d, *J* =8.0 Hz, pyridine-H), 8.42-8.44 (1H, d, *J* =8.0 Hz, pyridine-H), 8.71-8.72 (1H, d, *J* =4.0 Hz, pyridine-H), 10.68 (1H, s, NH). IR (KBr) m/cm^{-1}: 3312, 3035, 1695, 1602, 1583, 1176, 1080. MS m/z (ESI): 286 [M+H]+. Purity: 99.2% by HPLC (MeOH/0.1% TEA 85:15 (v/v); t_R(min). 2.08

Table 2. Kinetic parameters of AChE inhibited by compound 7a

C/μM	Michaelis-Menten equation	K_m/mM	V_{max}/ΔAmin^{-1}	Ki/μM	Ki'/μM
0	1/v=167.80/[S]+ 36.51	4.60	0.027		
0.85	1/v=240.12/[S]+ 60.10	4.00	0.017	4.69	3.28
1.70	1/v=271.48/[S]+ 70.66	3.84	0.014		
3.41	1/v=313.56/[S]+ 84.29	3.72	0.012		

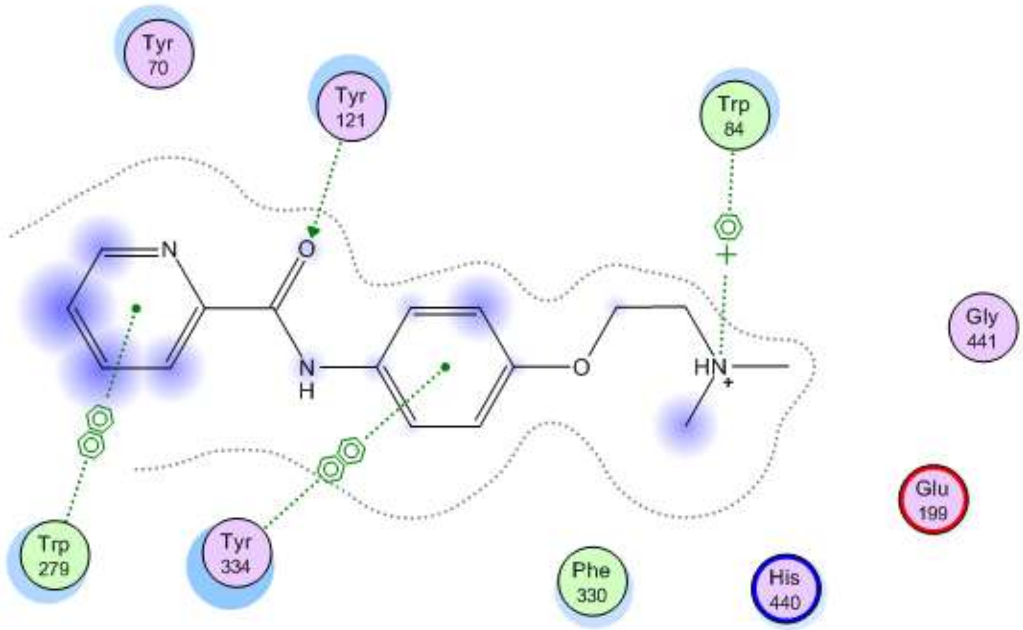
Tyr 70
Tyr 121
Trp 84
N
O
N H
O
HN+
Gly 441
Glu 199
Trp 279
Tyr 334
Phe 330
His 440

（A）

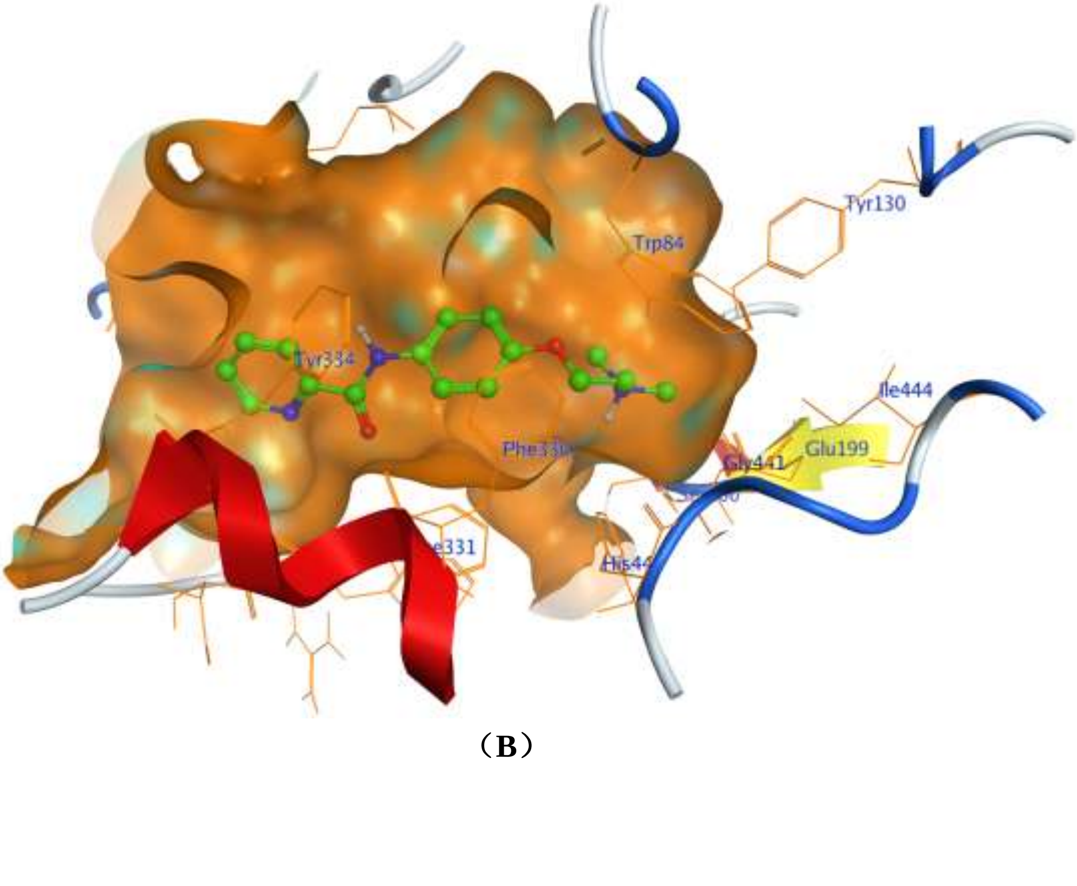
Tyr130
Trp84
Tyr334
Ile444
Glu199
Gly441

（B）

Figure 2. Molecular modeling of compound **7a** with AChE (**A, B**) and BChE (**C, D**) , generated with MOE.

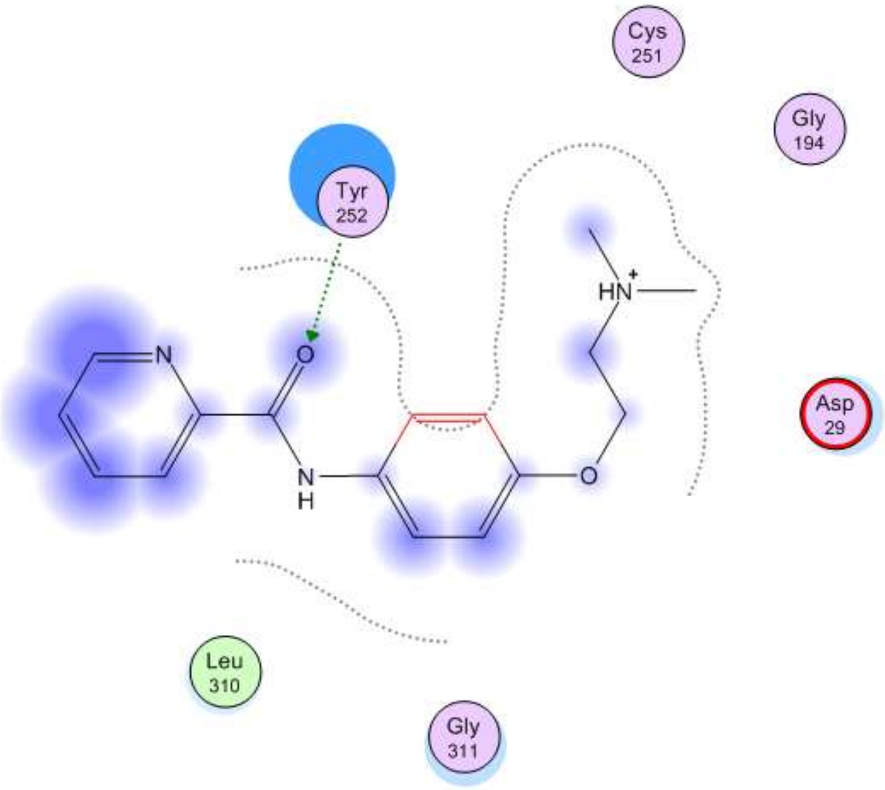

（**C**）

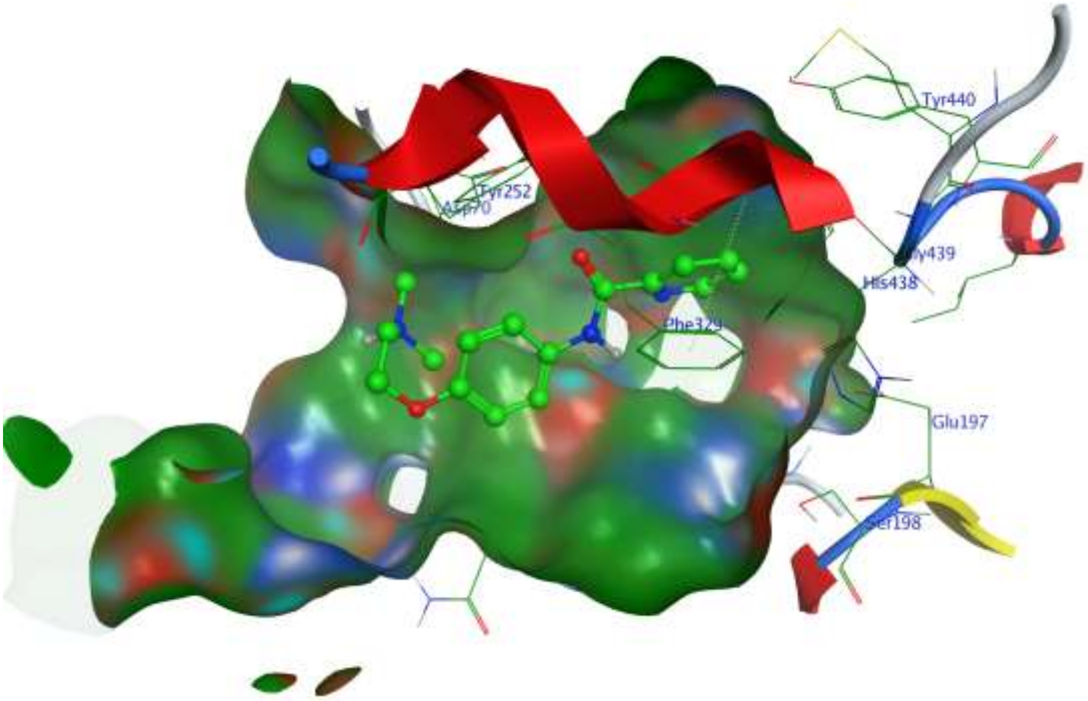

（**D**）

Figure 2. Molecular modeling of compound **7a** with AChE (**A, B**) and BChE (**C, D**) , generated with MOE.

Table 3 Molecular docking parameters of compound 7a binds to AChE and BChE, respectively.

	Amino acids sites	Action types	Distance(Å)	Energy (kcal.mol^{-1})
AChE	Trp84	π-cation	3.92	-22.7507
	Trp279	π - π	4.12	
	Tyr334	π - π	3.89	
BChE	Tyr252	Sidechain receptor	4.43	-16.6987
